# The Fraudulent Diploma Man

**Published:** 1880-12

**Authors:** 


*The Fraudulent Diploma Man.*—Dr. Buchanan, of Philadelphia, whose arrest has been published, was tried on a charge of conspiring to defraud, but was acquitted on the ground that the diplomas he sold were known to be worthless by those purchasing them. He was tried however on another indictment, and fined and imprisoned for a few months. The fraudulent diploma business will probably not be resumed by him, and it is to be hoped that it is effectually broken up.

